# Early‐Life Exposures and Risk of Multiple Myeloma: A Population‐Based Case–Control Study in Australia

**DOI:** 10.1002/ijc.70539

**Published:** 2026-05-14

**Authors:** Zhuoyu Sun, Julie K. Bassett, Simon Cheah, Fiona J. Bruinsma, Wendy Cozen, Simon J. Harrison, H. Miles Prince, Nicole Wong Doo, Graham G. Giles, Roger L. Milne, Brigid M. Lynch

**Affiliations:** ^1^ Department of Epidemiology and Biostatistics, School of Public Health Tianjin Medical University Tianjin China; ^2^ Cancer Epidemiology Division Cancer Council Victoria Melbourne Australia; ^3^ Centre for Epidemiology and Biostatistics, Melbourne School of Population and Global Health The University of Melbourne Melbourne Australia; ^4^ Burnet Institute Melbourne Australia; ^5^ University of California Irvine California USA; ^6^ Sir Peter MacCallum Department of Oncology The University of Melbourne Melbourne Australia; ^7^ Clinical Haematology Peter MacCallum Cancer Centre and Royal Melbourne Hospital Melbourne Australia; ^8^ Epworth Healthcare Melbourne Australia; ^9^ Concord Clinical School University of Sydney Sydney Australia; ^10^ Precision Medicine, School of Clinical Sciences at Monash Health Monash University Melbourne Australia

**Keywords:** breastfeeding, childhood environment, immune development, maternal smoking, pet ownership

## Abstract

Multiple myeloma (MM) is a hematologic malignancy with few known modifiable risk factors. Early‐life exposures may influence immune system development and, in turn, affect cancer susceptibility. We examined whether exposures while in utero or during early childhood were associated with MM risk. We conducted a population‐ and family‐based case–control study with 782 MM cases and 1121 controls. Early‐life exposures including maternal smoking, being breastfed, childhood growth patterns, household living arrangements, and pet ownership were self‐reported. Multivariable logistic regression models, adjusted for age at enrolment, sex, country of birth, socioeconomic position, rural–urban residence and birth weight, were fitted using multiple imputation by chained equations to handle missing data. Living in a home with five or more children was associated with a lower risk of MM (OR = 0.57, 95% CI: 0.38–0.86). Sharing a bedroom before age 11 years (OR = 0.80, 95% CI: 0.64–1.00) and pet ownership in early childhood (OR = 0.76, 95% CI: 0.60–0.98) were also inversely associated with MM risk. No clear associations were observed for maternal smoking during pregnancy (OR = 1.09, 95% CI: 0.79–1.51), being breastfed (OR = 0.96, 95% CI: 0.72–1.28), or height relative to peers at age 7 or 11 (7 years: OR = 1.10, 95% CI: 0.86–1.41; 11 years: OR = 1.06, 95% CI: 0.83–1.34). Our findings support the hypothesis that early‐life immune stimulation through environmental exposures may reduce the risk of MM. Further studies are needed to elucidate the biological mechanisms underlying these associations and to explore potential preventive strategies.

## Abstract (highlights)

What's new?

Early‐life exposures may influence immune system development and, in turn, affect cancer susceptibility. In this population‐based case‐control study, the authors examined whether in utero or early childhood exposures were associated with the risk of developing multiple myeloma in adulthood. Childhood exposures associated with greater microbial diversity such as living with five or more children, sharing a bedroom before age 11 years, and pet ownership in early childhood were associated with a lower risk of multiple myeloma. This work contributes to a more nuanced understanding of multiple myeloma etiology and highlights the importance of considering early‐life exposures in cancer prevention research.

## Graphical abstract

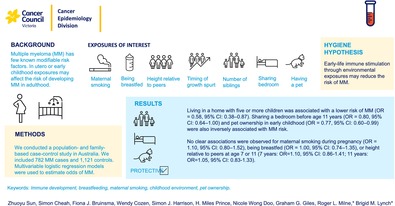

**Abbreviations**
CI: confidence interval
CONFIRM: Consortium for the Investigation of Renal Malignancies
EMMA: Epidemiology of Multiple Myeloma in Australia
MGUS: monoclonal gammopathy of undetermined significance
MICE: multiple imputation by chained equations
MM: multiple myeloma
OR: odds ratio
SEIFA: socioeconomic index for areas

## 1 Introduction

Multiple myeloma (MM) is a plasma cell malignancy arising from clonal proliferation within the bone marrow, accounting for 10%–15% of hematologic cancers globally. Despite recent advances in therapy, MM remains largely incurable, emphasizing the need for better understanding of its etiology. While several non‐modifiable risk factors such as older age, male sex, African ancestry, and family history are well‐established, few modifiable exposures have been identified [1, 2].

Recent research has focused on the long‐term influence of early‐life exposures on immune function and subsequent cancer risk. Exposures such as being breastfed and maternal smoking during pregnancy have been shown to modulate immune development, potentially affecting long‐term cancer susceptibility. Being breastfed has been found to be associated with a reduced risk of certain cancers, such as childhood leukemia, and may mitigate the negative impact of maternal smoking on lung cancer mortality in adulthood [3, 4]. These protective effects are thought to result from the transfer of immunologically active components, such as immunoglobulins and cytokines, which enhance immune maturation. However, large‐scale epidemiologic studies have found no significant association between being breastfed in infancy and the risk of adult cancers, including leukemia, lymphoma, or MM [5, 6].

Conversely, maternal smoking during pregnancy has been linked to impaired fetal immune development and an increased risk of certain childhood cancers, such as central nervous system cancers, although no clear association has been observed with childhood leukemia [7]. Smoking alters the composition of breast milk, including its immune properties, lipid content, and nutritional profile, potentially compromising the infant's immune development and increasing later susceptibility to disease [8, 9]. However, a recent systematic review and meta‐analysis of cohort studies suggested that maternal smoking during pregnancy may have minimal overall impact on cancer incidence in offspring [10]. The specific relationships between exposure to maternal smoking and being breastfed as a child and MM risk remain insufficiently understood and warrant further investigation.

Early microbial exposures, including those obtained through pet ownership or sharing a bedroom with siblings or other children, may influence immune programming and potentially reduce risk of immune‐related malignancies. For instance, early exposure to household pets has been linked to alterations in microbiome composition and immune regulation, with some studies suggesting a reduced risk of allergic diseases and possible benefits for immune function [11]. Similarly, factors such as birth order and sibship size have been found to be associated with lymphoma risk, with reduced risk for individuals with later birth order and more siblings [2]. Attendance at daycare during the first year of life has also been reported to be associated with a lower risk of asthma, likely due to gene–environment interactions shaping early immune phenotypes [12]. These observations support the hygiene hypothesis, which posits that exposure to infections in early childhood fosters immune development and may reduce the risk of certain cancers [13]. The potential association between early‐life exposures and immune‐related malignancies, including MM, is an area of growing interest that may reveal novel strategies for prevention.

To build upon previous findings, we aimed to examine associations between early‐life exposures and the risk of developing MM in adulthood, utilizing a population‐based case–control study. This investigation included a diverse range of exposures, from maternal health behaviors to early childhood environment, to identify potentially modifiable risk factors in the etiology of MM.

## 2 Materials and Methods

### 2.1 Study Sample

Participants were recruited to the Epidemiology of Multiple Myeloma in Australia (EMMA) study, designed to investigate genetic and environmental contributors to MM. Details of the recruitment process and eligibility criteria have been described elsewhere [14]. Briefly, newly diagnosed MM cases were recruited between 2010 and 2016 in Victoria and between 2013 and 2016 in New South Wales (NSW). Eligible cases were aged 20–74 years at diagnosis and had a histologically confirmed diagnosis of MM (ICD‐O‐3: M9732/3), a clinical diagnosis of monoclonal gammopathy of undetermined significance (MGUS), or related plasma cell disorders such as smoldering myeloma. Cases were recruited within 12 months of diagnosis, were residents of Victoria or NSW, and able to complete questionnaires in English. Once cases had agreed to participate, they were asked to nominate either the spouse/partner or a sibling (ideally a sibling closest in age to the case of the same sex, although this was not essential) without a history of hematologic malignancy and who were able to complete questionnaires in English. Additional controls were included from the Consortium for the Investigation of Renal Malignancies (CONFIRM) study, which applied a similar study design, based on cases with renal cancer in Victoria and Queensland [15]. Controls from the CONFIRM study were either siblings or spouses of the renal cancer cases.

For the main analyses, all non‐MM cases (including MGUS and related plasma cell disorders such as smoldering myeloma) were excluded to focus on incident MM and avoid diagnostic heterogeneity. Sibling controls (from the EMMA study) were also excluded to reduce bias from shared early‐life exposures. CONFIRM sibling controls were retained, as they were unrelated to the EMMA cases.

### 2.2 Exposure and Confounder Assessment

Consenting participants completed self‐administered questionnaires covering health and lifestyle, family and medical history, and residential history. The early‐life exposures of interest included: maternal smoking during pregnancy (yes/no), being breastfed as an infant (yes/no), height at age 7 and 11 years relative to peers (shorter, about the same, taller), timing of growth spurt during teenage years (before most others your age, about the same, later than most others), number of children lived with before age 11 (0, 1, 2, 3, 4, ≥ 5), shared a bedroom before age 11 years (yes/no), pet ownership before age 11 years (yes/no). Birth weight in kilograms was collected but not used as the key exposure variable due to the high percentage of missing data (49%).

Covariates for multivariable models were selected based on existing literature and causal diagram analysis (Figure S1A–E). All models were adjusted for age at enrollment (continuous) and sex (male/female). Other potential confounders included country of birth (Australia/New Zealand, Europe, other), socioeconomic position measured using the socioeconomic index for areas (SEIFA) index of relative socioeconomic disadvantage score (1 = most disadvantaged to 5 = most advantaged) and rural–urban residence (major city, regional/remote area; available for EMMA participants only). Age at enrollment was used as a proxy for birth cohort effect; for example, maternal smoking would have been more commonly reported by cases and controls born during the 1970s, when female smoking rates in Australia peaked.

### 2.3 Statistical Analysis

Multiple imputation was used to address missing values in covariates included in any analytical model using the multiple imputation by chained equations (MICE) approach. This method involves variable‐specific imputation models: linear regression for continuous variables, ordinal logistic regression for ordered categorical variables, multinomial logistic regression for nominal categorical variables, and predictive mean matching for semi‐continuous variables [16, 17, 18, 19]. To avoid perfect prediction issues in categorical variables, imputation was performed using augmented data [17]. A total of 50 imputed datasets were generated using the *mi* suite in Stata. Estimates from each dataset were then combined using Rubin's rules to obtain final pooled estimates and associated standard errors [20].

Descriptive statistics were calculated for cases and controls. Multivariable logistic regression was performed to estimate adjusted odds ratios (ORs) and 95% confidence intervals (CIs) for associations between early‐life exposures and MM risk, adjusting for potential confounders shown in directed acyclic graphs (see Figure S1A–E).

Several sensitivity analyses were performed to assess the robustness of the findings: (1) Restriction to EMMA participants only: to evaluate whether inclusion of CONFIRM controls (originally recruited for a renal cancer study) influenced the results. (2) Restriction of CONFIRM controls to Victoria only: to address potential geographic differences, given that a slightly larger proportion of MM cases were from Victoria. (3) Complete‐case analysis: to examine whether missing data influenced results and to compare with the main MICE‐imputed findings. We also additionally adjusted all models for marital status, to account for possible systematic differences between partnered and unpartnered people. All statistical tests were two‐sided and *p*‐values < 0.05 were considered statistically significant. All analyses were performed using Stata 16.0 (StataCorp LLC, College Station, TX, USA).

## 3 Results

### 3.1 Participant Characteristics

**FIGURE 1:**
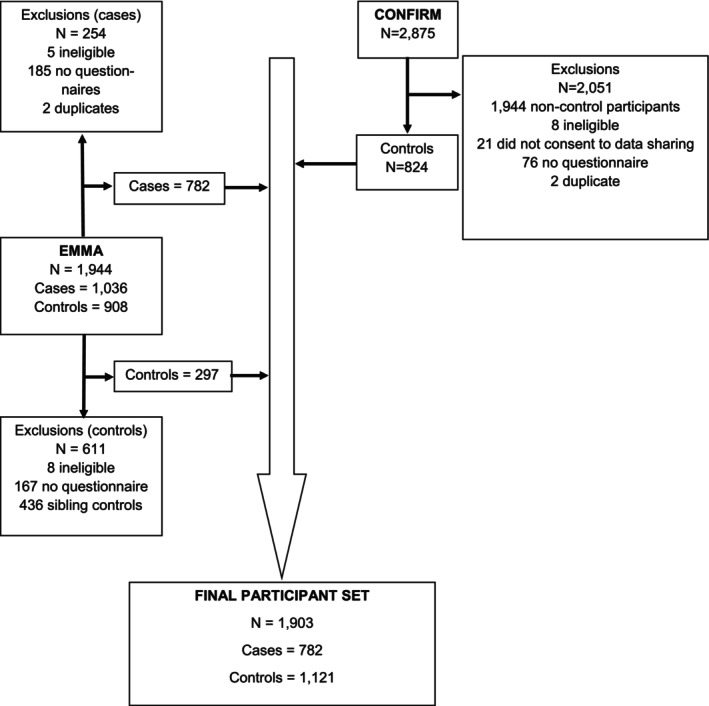
Flowchart of sample selection for analysis.

Figure 1 shows how cases and controls were selected for this study. A total of 1903 participants were included in the main analyses, comprising 782 cases and 1121 controls (297 from EMMA and 824 from CONFIRM). Compared with controls, cases were slightly older at enrollment (mean 63.1 ± [standard deviation] 8.4 years, EMMA controls: 62.2 ± 8.5, CONFIRM controls: 59.9 ± 9.8) and a higher proportion of cases were male (57.8%) compared with EMMA (33.7%) or CONFIRM controls (37.4%). A larger share of cases resided in Victoria (67.8%) than controls (63.3% EMMA, 53.5% CONFIRM). The proportion of participants born outside Australia/New Zealand was similar for cases (25.7%) and EMMA controls (28.2%), both notably higher than in CONFIRM controls (15.2%). Cases were more likely to live in socioeconomically disadvantaged areas (34.9%) than either EMMA (30.3%) or CONFIRM controls (31.3%). In contrast, CONFIRM controls had a higher prevalence of obesity (31.7%) than EMMA cases (21.9%) or EMMA controls (18.9%), and a higher rate of current smoking (8.6% vs. 7.8% of EMMA cases and 7.1% of EMMA controls). There were considerable differences in marital status: 79% of cases were partnered, while 96% of EMMA controls and 86% of CONFIRM controls were partnered. The number of children per household during early life was essentially the same across cases and controls (Table 1).

**TABLE 1:** Characteristics of all cases and controls.

Characteristics	Cases (*N* = 782)	EMMA controls (*N* = 297)	CONFIRM controls (*N* = 824)
Age at H & L Qx completion, mean (SD)	63.1 (8.4)	62.2 (8.5)	59.9 (9.8)
Age at diagnosis, mean (SD)	62.2 (8.5)	—	—
Sex, *N* (%)			
Female	330 (42.2)	197 (66.3)	516 (62.6)
Male	452 (57.8)	100 (33.7)	308 (37.4)
State, *N* (%)			
Victoria	530 (67.8)	188 (63.3)	441 (53.5)
New South Wales	252 (32.2)	109 (36.7)	0 (0)
Queensland	0 (0)	0 (0)	383 (46.5)
Country of birth, *N* (%)			
Australia or New Zealand	577 (73.8)	213 (71.7)	698 (84.7)
Europe (including UK)	133 (17.0)	53 (17.8)	97 (11.8)
Other	68 (8.7)	31 (10.4)	28 (3.4)
Missing	4 (0.5)	0 (0)	1 (0.1)
BMI (kg/m^2^), mean (SD)	27.2 (4.7)	26.7 (4.9)	28.1 (5.9)
BMI (kg/m^2^), *N* (%)			
< 18.5	7 (0.9)	3 (1.0)	6 (0.7)
18.5–24.9	259 (33.1)	100 (33.7)	249 (30.2)
25–29.9	321 (41.0)	125 (42.1)	283 (34.3)
30+	171 (21.9)	56 (18.9)	261 (31.7)
Missing	24 (3.1)	13 (4.4)	25 (3.0)
Alcohol consumption, *N* (%)			
Non‐drinker	128 (16.4)	45 (15.2)	128 (15.5)
Moderate (≤ 20 g) drinker	455 (58.2)	177 (59.6)	516 (62.6)
Heavy (> 20 g) drinker	162 (20.7)	62 (20.9)	166 (20.1)
Missing	37 (4.7)	13 (4.4)	14 (1.7)
Smoking status, *N* (%)			
Never smoker	428 (54.7)	180 (60.6)	465 (56.4)
Former smoker	289 (37.0)	96 (32.3)	268 (32.5)
Current smoker	61 (7.8)	21 (7.1)	71 (8.6)
Missing	4 (0.5)	0 (0)	20 (2.4)
SEIFA, *N* (%)			
1 = most disadvantage	123 (15.7)	41 (13.8)	130 (15.8)
2	150 (19.2)	49 (16.5)	136 (16.5)
3	149 (19.1)	49 (16.5)	182 (22.1)
4	150 (19.2)	63 (21.2)	154 (18.7)
5 = most advantage	209 (26.7)	94 (31.6)	217 (26.3)
Missing	1 (0.1)	1 (0.3)	5 (0.6)
Marital status, *N* (%)			
Never/widowed/divorced/separated	162 (20.7)	12 (4.0)	113 (13.7)
Married/de facto	615 (78.6)	285 (96.0)	710 (86.2)
Missing	5 (0.6)	0 (0)	1 (0.1)
Children in household, median (25th, 75th percentile)	3 (2, 4)	3 (2, 5)	3 (2, 5)

### 3.2 Associations Between Early‐Life Exposures and MM Risk

Table 2 presents the differences in early‐life exposures observed between cases and controls. Cases less commonly reported pet ownership before age 11 years than controls (76.9% vs. 78.0%) and were less likely to have grown up in larger families (living with ≥ 5 children: 12.5% vs. 14.1%). In multivariable logistic regression models, several early‐life exposures were associated with MM risk. Individuals who reported living with five or more children had a significantly lower risk of MM (OR = 0.57, 95% CI: 0.38–0.86). Sharing a bedroom before age 11 years was also associated with a lower risk of MM (OR = 0.80, 95% CI: 0.64–1.00), as was pet ownership before age 11 years (OR = 0.76, 95% CI: 0.60–0.98).

**TABLE 2:** Odds ratios (95% CIs) for early life exposures and MM risk among all cases and controls.

Exposure	Cases, *n* (%)	Controls, *n* (%)	OR	(95% CI)
Maternal smoking in pregnancy^a^				
No	605 (77.4)	820 (73.1)	1.00	
Yes	91 (11.6)	110 (9.8)	1.09	(0.79, 1.51)
Missing	86 (11.0)	191 (17.0)		
Breastfeeding^a^				
No	98 (12.5)	156 (13.9)	1.00	
Yes	514 (65.7)	664 (59.2)	0.96	(0.72, 1.28)
Missing	170 (21.7)	301 (26.9)		
Height compared to other children at age 7^b^				
About the same	446 (57.0)	597 (53.3)	1.00	
Shorter	152 (19.4)	244 (21.8)	0.86	(0.67, 1.10)
Taller	175 (22.4)	209 (18.6)	1.10	(0.86, 1.41)
Missing	9 (1.2)	71 (6.3)		
Height compared to other children at age 11^b^				
About the same	409 (52.3)	542 (48.3)	1.00	
Shorter	161 (20.6)	250 (22.3)	0.88	(0.68, 1.12)
Taller	203 (26.0)	258 (23.0)	1.06	(0.83, 1.34)
Missing	9 (1.2)	71 (6.3)		
Time of growth spurt during teenage years^b^				
About the same	599 (76.6)	794 (70.8)	1.00	
Before most others	71 (9.1)	110 (9.8)	0.92	(0.66, 1.29)
After most others	94 (12.0)	135 (12.0)	0.93	(0.69, 1.25)
Missing	18 (2.3)	82 (7.3)		
Number of children you lived with (excluding yourself)^a^				
0	89 (11.4)	79 (7.0)	1.00	
1	155 (19.8)	211 (18.8)	0.76	(0.52, 1.11)
2	185 (23.7)	280 (25.0)	0.71	(0.49, 1.03)
3	160 (20.5)	202 (18.0)	0.91	(0.62, 1.34)
4	88 (11.3)	123 (11.0)	0.75	(0.49, 1.14)
5+	98 (12.5)	158 (14.1)	0.57	(0.38, 0.86)
Missing	7 (0.90)	68 (6.1)		
Shared a bedroom before 11 years old^a^				
No	208 (26.6)	235 (21.0)	1.00	
Yes	568 (72.6)	818 (73.0)	0.80	(0.64, 1.00)
Missing	6 (0.77)	68 (6.1)		
Contact with pets before 11 years old^a^				
No	175 (22.4)	173 (15.4)	1.00	
Yes	601 (76.9)	874 (78.0)	0.76	(0.60, 0.98)
Missing	6 (0.77)	74 (6.6)		

^a^ OR adjusted for sex, age at enrolment, country of birth, and SEIFA.

^b^ OR adjusted for sex, age at enrolment, country of birth, SEIFA, and birth weight.

By contrast, no clear associations were observed for maternal smoking during pregnancy (OR = 1.09, 95% CI: 0.79–1.51), being breastfed (OR = 0.96, 95% CI: 0.72–1.28) in infancy. Self‐reported height relative to peers, whether taller at age 7 years (OR = 1.10, 95% CI: 0.86–1.41) or age 11 years (OR = 1.06, 95% CI: 0.83–1.34), did not appear to influence MM risk. Similarly, reporting an earlier timing of growth spurts during adolescence was not associated with MM risk (OR = 0.93, 95% CI: 0.69–1.25), compared with reporting onset at about the same time as peers.

### 3.3 Sensitivity Analyses

Sensitivity analyses, including those restricted to EMMA controls only (Table S1) and complete‐case datasets (Tables S2 and S3), gave results consistent with those from the primary analyses for all exposures. The one exception was for being shorter than peers at age seven in the complete case analysis (Table S2, OR = 0.66, 95% CI: 0.47–0.94) and the complete case analysis restricted to EMMA participants only (Table S3, OR = 0.60, 95% CI: 0.37–0.99). Moreover, sensitivity analyses restricting CONFIRM controls to Victoria only (Table S4) produced effect estimates similar to the primary analysis, suggesting our findings are robust to this potential source of bias. Additional adjustment for marital status did not change results in any substantive way (Tables S5 and S6).

## 4 Discussion

Our study provides evidence that early‐life environmental exposures are associated with the risk of developing MM in adulthood. Specifically, exposures reflecting greater immune stimulation in early childhood, such as living with more than five children, sharing a bedroom, and early contact with pets, were associated with a reduced risk. These findings underscore the potential long‐term immunologic impact of early‐life exposures, an area that has been seldom explored in MM.

A key finding was the inverse association between living with five or more other children at home and MM risk. Growing up in a larger household likely increases childhood exposure to common infections and non‐pathogenic microbes, which can stimulate immune system maturation. This is consistent with prior epidemiologic evidence from allergic and infectious disease research: for example, having older siblings and household pets has each been associated with reduced risk of childhood allergic diseases [21, 22]. Here, we extend that concept to MM by showing an analogous protective pattern. Likewise, we found sharing a bedroom with other children (often a correlate of larger family size) to be associated with reduced MM risk. Early contact with pets also showed a protective association: pet ownership in childhood was linked to a 23% lower MM risk. These results point toward a role for animal‐associated microbial exposures in shaping immune development, since household pets can markedly alter the infant gut microbiome and enhance colonization with immune‐stimulating bacteria [22].

These consistent associations support the “hygiene hypothesis,” which proposes that reduced exposure to microbes in early life, due to increasingly sanitized environments, may impair immune system maturation. This imbalance is thought to increase susceptibility to immune‐related diseases, including allergies, autoimmune conditions, and some cancers [13, 23, 24]. Early‐life immune education, particularly exposure to a diverse range of antigens, is critical for the development of a balanced Type 1 Helper/Type 2 Helper cell immune response [25, 26, 27]. A lack of such stimulation may result in immune dysregulation, chronic inflammation, or aberrant immune cell proliferation [25, 28, 29].

Mechanistically, MM is characterized by dysregulated plasma cell proliferation in the context of chronic immune stimulation. Early‐life exposures that broaden immune contacts may help calibrate immune responses and prevent pro‐inflammatory dysregulation. For instance, Dhodapkar [30] emphasizes that MM and its precursor, MGUS, likely arise in the setting of chronic immune activation. It is plausible that richer microbial exposures in childhood could foster immune regulation that delays or mitigates this chronic activation cascade. In line with this idea, we speculate that our observed protective associations may reflect more specific effects on MM pathogenesis, for example, slowing progression from MGUS to overt myeloma, rather than a general cancer‐prevention effect. Such a pathway could be analogous to findings in other hematologic diseases, where early immune challenges seem to promote immunologic maturation and reduce the later development of disorders [24, 31, 32]. In fact, recent reviews highlight that maintaining a diverse infant gut microbiota (aided by factors like pets and breastfeeding) is crucial for healthy immune development and prevention of immune‐mediated diseases [31]. Our results for pet ownership and bedroom sharing are concordant with this body of evidence: childhood pet exposure has been shown to enrich gut bacterial genera linked to lower allergy risk, and a well‐colonized gut microbiota is thought to train the immune system away from pro‐inflammatory responses [22, 33].

Despite its well‐established role in supporting immune development [34, 35], being breastfed was not associated with MM risk in our study. This is consistent with previous literature: breastfeeding provides bioactive components that support infant health [35], but epidemiologic studies of adult cancer have not linked it strongly to hematologic malignancies [5, 6]. For instance, a large United Kingdom cohort study of about 550,000 middle‐aged women (the Million Women Study) compared those who had been breastfed in infancy with those who had not. Aside from a modest increased risk of colorectal cancer among women who were breastfed, there were no significant associations with any other cancers—including leukemia, lymphoma, or MM [6]. Similarly, no association was observed between maternal smoking during pregnancy and MM, consistent with prior meta‐analyses suggesting minimal effects of maternal smoking during pregnancy on overall cancer incidence [10]. Another meta‐analysis of 40 observational studies also found no evidence of association between tobacco use and MM in adulthood [36]. These findings suggest that smoking's impact on non‐smoking‐related cancers (including MM) may be minimal, and our results support that conclusion.

Childhood growth patterns, particularly height relative to peers, have been linked to the risk of various cancers, including breast, colon, thyroid, endometrial, and ovarian cancer [1, 37, 38, 39, 40, 41, 42]. However, no evidence of association was found between childhood height or adolescent growth spurts and MM risk in the main analysis using MICE. Interestingly, a sensitivity analysis using complete‐case data revealed an inverse association between being shorter at age 7 years and MM risk. This pattern was observed both in the EMMA‐only analysis and in the combined dataset. Given that MICE generally offer better statistical efficiency and reduce bias (assuming data are missing at random), this complete‐case finding should be interpreted with caution. It may reflect subgroup effects, residual confounding, or a weak association sensitive to data availability. The contrasting finding between MICE and complete‐case analyses emphasizes the importance of using robust statistical methods when handling missing data.

Several limitations are present in this study. As a retrospective case–control analysis, exposure data were self‐reported and thus subject to potential recall bias. Some early‐life exposures (e.g., sibship size, bedroom sharing, and pet ownership) are likely to be recalled with reasonable accuracy even decades later, whereas others (notably maternal smoking during pregnancy and breastfeeding) may be difficult to remember reliably. If misclassification occurred, it might not have been nondifferential: it is possible that cases and controls recall certain past exposures differently. Differential recall could bias the results in either direction (toward or away from the null), making the net effect unpredictable. We therefore interpret our findings with caution, acknowledging that recall bias could have influenced some associations. Selection bias due to non‐participation, particularly if related to disease severity or missing data, may also be a concern; however, this was addressed through the use of MICE, which reduces bias by incorporating uncertainty from missing data. Because case participants were not required to have siblings, whereas some control participants were selected specifically as siblings, residual systematic differences in family structure may persist. This is particularly important given that the study examines early‐life exposures related to sibship. We also acknowledge that a slightly larger proportion of cases than controls were from Victoria. This geographic imbalance could potentially introduce regional confounding. However, results from sensitivity analyses restricted to Victorian controls yielded similar effect estimates, suggesting that the observed associations were not materially influenced by geographic differences. This consistency supports the validity of the combined analysis using both EMMA and CONFIRM controls. There is a possibility that residual confounding, such as parental socioeconomic status, aspects of the home environment, or unmeasured familial risk, influenced the findings, although adjustments were made for key covariates based on causal diagram analysis. Information on paternal smoking or other paternal exposures during pregnancy was not collected, which may have introduced unmeasured confounding in analyses of maternal smoking. Ethnic diversity was limited, and findings may not generalize to populations with different genetic backgrounds. Additionally, the lack of mechanistic data limits the ability to confirm biological pathways implicated in the observed associations.

Strengths of the study include its large, population‐based case–control design, the inclusion of incident MM cases, and the use of two distinct control sources. Importantly, sibling controls from EMMA were excluded to minimize bias from shared early‐life environments. This ensured that controls were comparable to cases in terms of family exposure context. The detailed assessment of early‐life exposures, an often‐overlooked period in epidemiologic research, adds depth to our understanding of MM risk factors. Moreover, the use of MICE to address missing data strengthened the internal validity of the findings. The consistency of results across multiple sensitivity analyses (including those restricted to Victoria controls and spouse/partner controls) reinforces the robustness of the conclusions.

## 5 Conclusion

In conclusion, our study provides important epidemiological evidence supporting a link between early‐life environmental exposures and the risk of developing MM. Specifically, childhood exposures associated with greater microbial diversity, such as living with five or more children, sharing a bedroom, and early contact with pets, were associated with a lower risk of MM. These findings are consistent with the hygiene hypothesis and suggest that immune modulation during early childhood may influence MM risk later in life. This work contributes to a more nuanced understanding of MM etiology and highlights the importance of considering early‐life exposures in cancer prevention research. Future studies, particularly longitudinal and mechanistic investigations, are warranted to elucidate the immunological pathways linking childhood exposures to MM development and to explore potential interventions that mimic these protective effects.

## Author Contributions

**Zhuoyu Sun:** conceptualization, formal analysis, writing – original draft, writing – review and editing, methodology. **Julie K. Bassett:** methodology, writing – review and editing, formal analysis. **Simon Cheah:** conceptualization, methodology, formal analysis, writing – original draft, writing – review and editing. **Fiona J. Bruinsma:** conceptualization, investigation, data curation, project administration, writing – review and editing. **Wendy Cozen:** investigation, funding acquisition, writing – review and editing. **Simon J. Harrison:** investigation, funding acquisition, writing – review and editing. **H. Miles Prince:** investigation, funding acquisition, writing – review and editing. **Nicole Wong Doo:** investigation, funding acquisition, writing – review and editing. **Graham G. Giles:** conceptualization, methodology, investigation, funding acquisition, writing – review and editing, project administration, resources. **Roger L. Milne:** resources, writing – original draft, writing – review and editing, methodology, supervision, conceptualization. **Brigid M. Lynch:** conceptualization, methodology, supervision, writing – original draft, writing – review and editing.

## Funding

This work was supported by the National Health and Medical Research Council Project Grant (Grant ID 1029885).

## Ethics Statement

All participants provided written informed consent. The study was approved by the Cancer Council Victoria Human Research Ethics Committee (CCV HREC ref. 1001) and the New South Wales Population and Health Services Research Ethics Committee (CCV HREC ref. 0912).

## Conflicts of Interest

The authors declare no conflicts of interest.

## Supporting information


**Figure S1:** Directed acyclic graphs.
**Table S1:** Sensitivity analysis for early life exposures and MM risk among EMMA cases and EMMA controls.
**Table S2:** Sensitivity analysis restricting CONFIRM controls to Victoria only for early life exposures and MM risk.
**Table S3:** Complete case analysis for early life exposures and MM risk among all cases and controls.
**Table S4:** Complete case analysis for early life exposures and MM risk among EMMA cases and EMMA controls.
**Table S5:** Sensitivity analysis for early life exposures and MM risk among EMMA cases and EMMA controls with additional adjustment for marital status.
**Table S6:** Sensitivity analysis restricting CONFIRM controls to Victoria only for early life exposures and MM risk with additional adjustment for marital status.

## Data Availability

Deidentified data that support the findings of this study are available from the corresponding author upon request.
