# Supplementary material for: Early‐Life Exposures and Risk of Multiple Myeloma: A Population‐Based Case–Control Study in Australia
Source: Int J Cancer. 2026 May 14;159(7):1697–706. doi: 10.1002/ijc.70539 (PMC13432434; doi:10.1002/ijc.70539)
Supplement: Supplementary file 1 — Figure S1: Directed acyclic graphs. Table S1: Sensitivity analysis for early life exposures and MM risk among EMMA cases and EMMA controls. Table S2: Sensitivity analysis restricting CONFIRM controls to Victoria only for early life exposures and MM risk. Table S3: Complete case analysis for early life exposures and MM risk among all cases and controls. Table S4: Complete case analysis for early life exposures and MM risk among EMMA cases and EMMA controls. Table S5: Sensitivity analysis for early life exposures and MM risk among EMMA cases and EMMA controls with additional adjustment for marital status. Table S6: Sensitivity analysis restricting CONFIRM controls to Victoria only for early life exposures and MM risk with additional adjustment for marital status. [file IJC-159-1697-s001.pdf]

Early-Life Exposures and Risk of Multiple Myeloma: A Population-based Case-Control Study in Australia

Zhuoyu Sun, Julie K. Bassett, Simon Cheah, Fiona J. Bruinsma, Wendy Cozen, Simon J. Harrison, H. Miles Prince, Nicole Wong Doo, Graham G. Giles, Roger L. Milne, Brigid M. Lynch

SUPPLEMENTARY MATERIAL

Table of contents

p. 2-4	Figures A – E	Directed acyclic graphs
p. 5	Table S1	Sensitivity analysis for early life exposures and MM risk among EMMA cases and EMMA controls
p. 6	Table S2	Sensitivity analysis restricting CONFIRM controls to Victoria only for early life exposures and MM risk
p. 7	Table S3	Complete case analysis for early life exposures and MM risk among all cases and controls
p. 8	Table S4	Complete case analysis for early life exposures and MM risk among EMMA cases and EMMA controls
p. 9	Table S5	Sensitivity analysis for early life exposures and MM risk among EMMA cases and EMMA controls with additional adjustment for marital status
p. 10	Table S6	Sensitivity analysis restricting CONFIRM controls to Victoria only for early life exposures and MM risk with additional adjustment for marital status

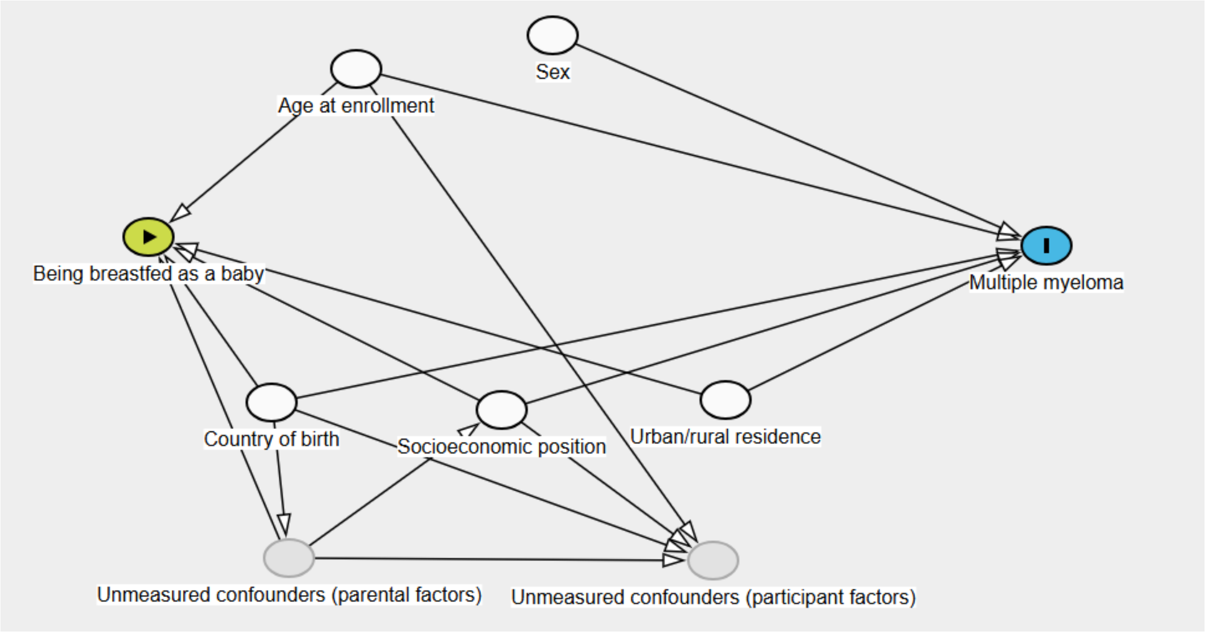

Figure (A): Directed acyclic graph for being breastfed and risk of multiple myeloma

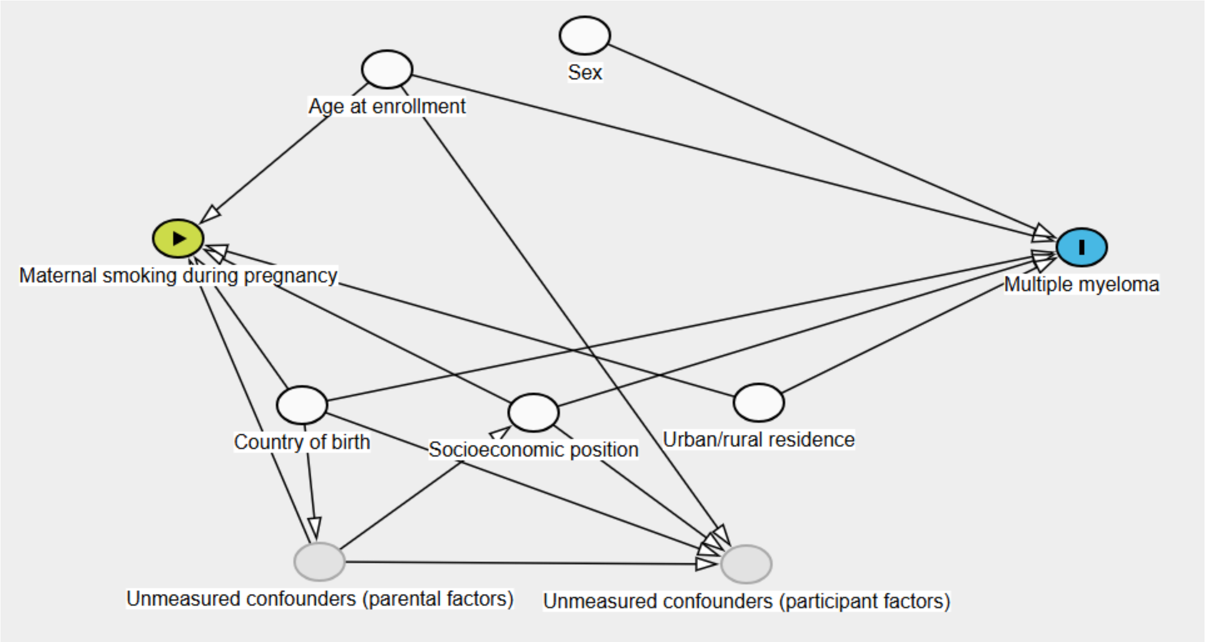

Figure (B): Directed acyclic graph for maternal smoking and risk of multiple myeloma

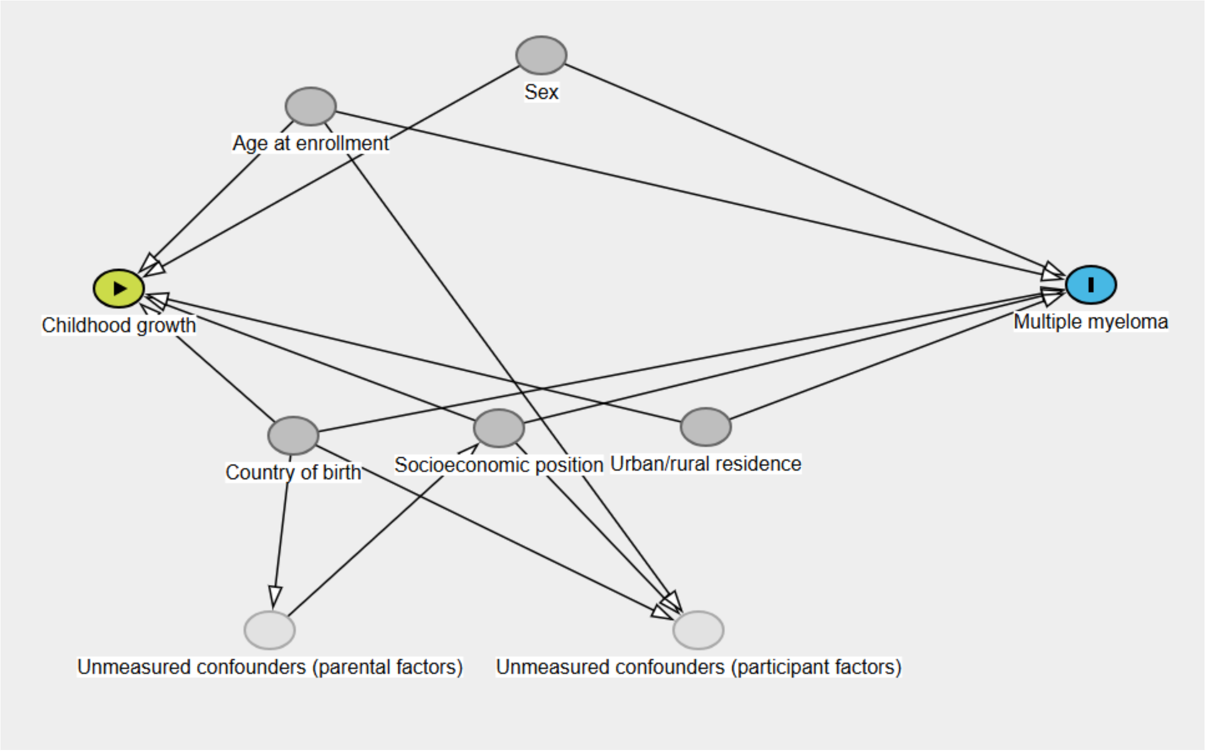

Figure (C): Directed acyclic graph for childhood growth and risk of multiple myeloma

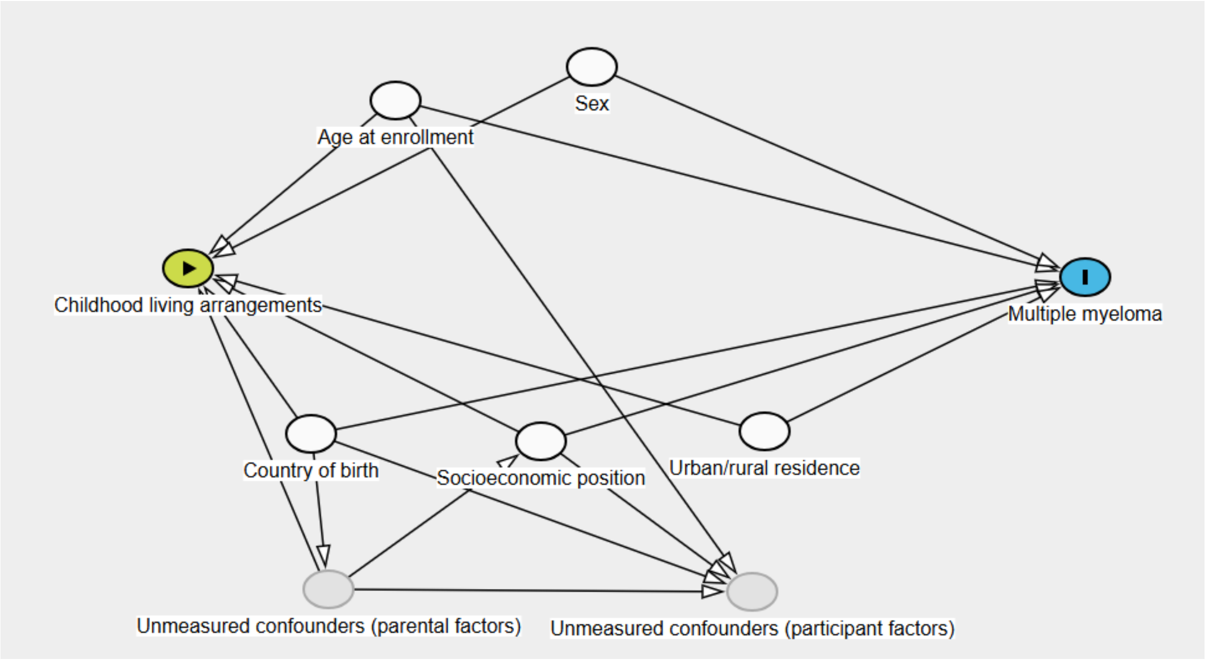

Figure (D): Directed acyclic graph for childhood living arrangements and risk of multiple myeloma

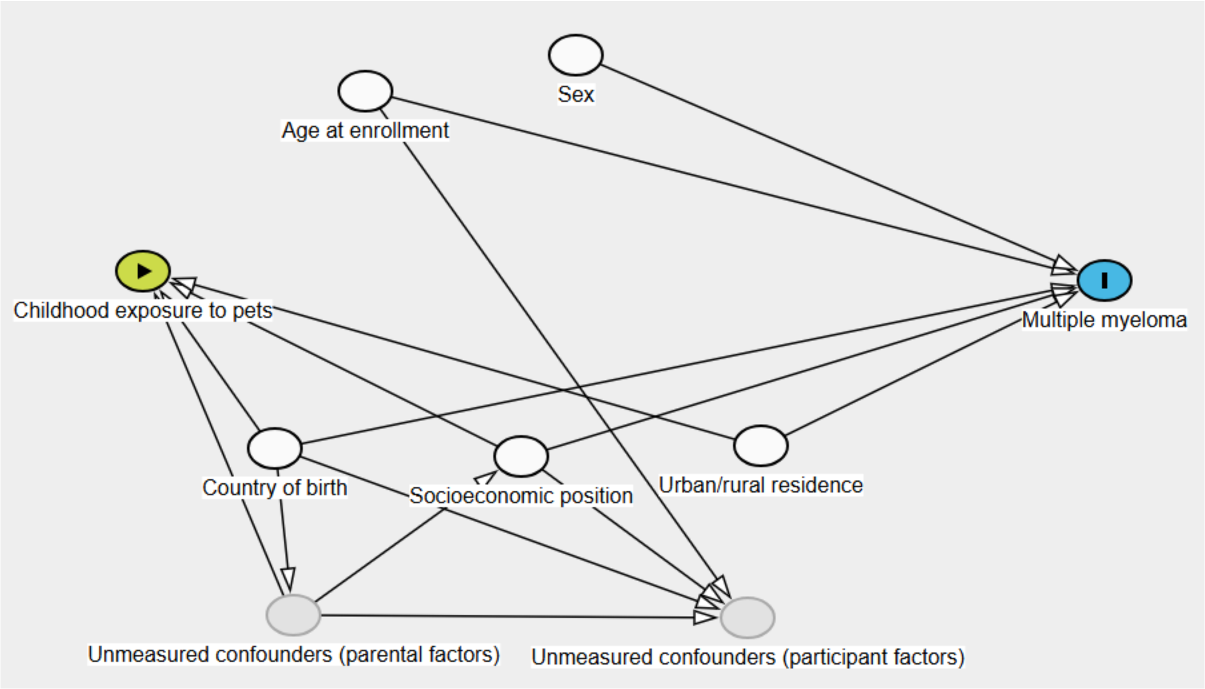

Figure (E): Directed acyclic graph for early life exposure to pets and risk of multiple myeloma

Table S1. Sensitivity analysis for early life exposures and MM risk among EMMA cases and EMMA controls

Exposure	Cases, n (%)	Controls, n (%)	OR	(95% CI)
Maternal smoking in pregnancy[a]				
No	605 (77.4)	224 (75.4)	1.00	
Yes	91 (11.6)	32 (10.8)	1.01	(0.65, 1.57)
Missing	86 (11.0)	41 (13.8)		
Breastfeeding[a]				
No	98 (12.5)	50 (16.8)	1.00	
Yes	514 (65.7)	197 (66.3)	1.22	(0.83, 1.81)
Missing	170 (21.7)	50 (16.8)		
Height compared to other children at age 7[b]				
About the same	446 (57.0)	152 (51.2)	1.00	
Shorter	152 (19.4)	68 (22.9)	0.77	(0.54, 1.10)
Taller	175 (22.4)	76 (25.6)	0.77	(0.55, 1.09)
Missing	9 (1.2)	1 (0.34)		
Height compared to other children at age 11[b]				
About the same	409 (52.3)	139 (46.8)	1.00	
Shorter	161 (20.6)	64 (21.5)	0.85	(0.59, 1.22)
Taller	203 (26.0)	93 (31.3)	0.72	(0.52, 1.00)
Missing	9 (1.2)	1 (0.34)		
Time of growth spurt during teenage years[b]				
About the same	599 (76.6)	227 (76.4)	1.00	
Before most others	71 (9.1)	33 (11.1)	0.90	(0.57, 1.42)
After most others	94 (12.0)	35 (11.8)	0.96	(0.62, 1.48)
Missing	18 (2.3)	2 (0.67)		
Number of children you lived with (excluding yourself) [a]				
0	89 (11.4)	26 (8.8)	1.00	
1	155 (19.8)	71 (23.9)	0.67	(0.39, 1.15)
2	185 (23.7)	76 (25.6)	0.70	(0.41, 1.19)
3	160 (20.5)	49 (16.5)	1.03	(0.59, 1.80)
4	88 (11.3)	32 (10.8)	0.80	(0.43, 1.47)
5+	98 (12.5)	43 (14.5)	0.68	(0.38, 1.22)
Missing	7 (0.90)	0 (0.00)		
Shared a bedroom before 11 years old[a]				
No	208 (26.6)	76 (25.6)	1.00	
Yes	568 (72.6)	220 (74.1)	0.95	(0.69, 1.30)
Missing	6 (0.77)	1 (0.34)		
Contact with pets before 11 years old[a]				
No	175 (22.4)	54 (18.2)	1.00	
Yes	601 (76.9)	242 (81.5)	0.72	(0.50, 1.03)
Missing	6 (0.77)	1 (0.34)		

[a] OR adjusted for sex, age at enrolment, sex, country of birth, SEIFA and rural-urban residence

[b] OR adjusted for sex, age at enrolment, country of birth, SEIFA, rural-urban residence and birth weight

Table S2. Complete case analysis for early life exposures and MM risk among all cases and controls

Exposure	Cases, n (%)	Controls, n (%)	OR	(95% CI)
Maternal smoking in pregnancy[a]				
No	605 (77.4)	820 (73.1)	1.00	
Yes	91 (11.6)	110 (9.8)	1.13	(0.83, 1.54)
Missing	86 (11.0)	191 (17.0)		
Breastfeeding[a]				
No	98 (12.5)	156 (13.9)	1.00	
Yes	514 (65.7)	664 (59.2)	0.99	(0.74, 1.32)
Missing	170 (21.7)	301 (26.9)		
Height compared to other children at age 7[b]				
About the same	446 (57.0)	597 (53.3)	1.00	
Shorter	152 (19.4)	244 (21.8)	0.66	(0.47, 0.94)
Taller	175 (22.4)	209 (18.6)	1.02	(0.73, 1.44)
Missing	9 (1.2)	71 (6.3)		
Height compared to other children at age 11[b]				
About the same	409 (52.3)	542 (48.3)	1.00	
Shorter	161 (20.6)	250 (22.3)	0.75	(0.53, 1.06)
Taller	203 (26.0)	258 (23.0)	1.08	(0.78, 1.50)
Missing	9 (1.2)	71 (6.3)		
Time of growth spurt during teenage years[b]				
About the same	599 (76.6)	794 (70.8)	1.00	
Before most others	71 (9.1)	110 (9.8)	1.18	(0.76, 1.82)
After most others	94 (12.0)	135 (12.0)	0.88	(0.59, 1.34)
Missing	18 (2.3)	82 (7.3)		
Number of children you lived with (excluding yourself) [a]				
0	89 (11.4)	79 (7.0)	1.00	
1	155 (19.8)	211 (18.8)	0.76	(0.52, 1.11)
2	185 (23.7)	280 (25.0)	0.71	(0.49, 1.04)
3	160 (20.5)	202 (18.0)	0.93	(0.63, 1.36)
4	88 (11.3)	123 (11.0)	0.73	(0.48, 1.12)
5+	98 (12.5)	158 (14.1)	0.58	(0.38, 0.88)
Missing	7 (0.90)	68 (6.1)		
Shared a bedroom before 11 years old[a]				
No	208 (26.6)	235 (21.0)	1.00	
Yes	568 (72.6)	818 (73.0)	0.80	(0.64, 1.00)
Missing	6 (0.77)	68 (6.1)		
Contact with pets before 11 years old[a]				
No	175 (22.4)	173 (15.4)	1.00	
Yes	601 (76.9)	874 (78.0)	0.76	(0.60, 0.98)
Missing	6 (0.77)	74 (6.6)		

a OR adjusted for sex, age at enrolment, sex, country of birth and SEIFA
b OR adjusted for sex, age at enrolment, country of birth, SEIFA, and birth weight

Table S3. Complete case analysis for early life exposures and MM risk among EMMA cases and EMMA controls

Exposure	Cases, n (%)	Controls, n (%)	OR	(95% CI)
Maternal smoking in pregnancy[a]				
No	605 (77.4)	224 (75.4)	1.00	
Yes	91 (11.6)	32 (10.8)	1.06	(0.67, 1.67)
Missing	86 (11.0)	41 (13.8)		
Breastfeeding[a]				
No	98 (12.5)	50 (16.8)	1.00	
Yes	514 (65.7)	197 (66.3)	1.29	(0.87, 1.92)
Missing	170 (21.7)	50 (16.8)		
Height compared to other children at age 7[b]				
About the same	446 (57.0)	152 (51.2)	1.00	
Shorter	152 (19.4)	68 (22.9)	0.60	(0.37, 0.99)
Taller	175 (22.4)	76 (25.6)	0.75	(0.46, 1.23)
Missing	9 (1.2)	1 (0.34)		
Height compared to other children at age 11[b]				
About the same	409 (52.3)	139 (46.8)	1.00	
Shorter	161 (20.6)	64 (21.5)	0.83	(0.50, 1.36)
Taller	203 (26.0)	93 (31.3)	0.82	(0.51, 1.32)
Missing	9 (1.2)	1 (0.34)		
Time of growth spurt during teenage years[b]				
About the same	599 (76.6)	227 (76.4)	1.00	
Before most others	71 (9.1)	33 (11.1)	1.10	(0.59, 2.03)
After most others	94 (12.0)	35 (11.8)	0.98	(0.53, 1.82)
Missing	18 (2.3)	2 (0.67)		
Number of children you lived with (excluding yourself) [a]				
0	89 (11.4)	26 (8.8)	1.00	
1	155 (19.8)	71 (23.9)	0.67	(0.39, 1.15)
2	185 (23.7)	76 (25.6)	0.72	(0.42, 1.22)
3	160 (20.5)	49 (16.5)	1.04	(0.59, 1.82)
4	88 (11.3)	32 (10.8)	0.79	(0.43, 1.47)
5+	98 (12.5)	43 (14.5)	0.68	(0.38, 1.23)
Missing	7 (0.90)	0 (0.00)		
Shared a bedroom before 11 years old[a]				
No	208 (26.6)	76 (25.6)	1.00	
Yes	568 (72.6)	220 (74.1)	0.95	(0.69, 1.31)
Missing	6 (0.77)	1 (0.34)		
Contact with pets before 11 years old[a]				
No	175 (22.4)	54 (18.2)	1.00	
Yes	601 (76.9)	242 (81.5)	0.72	(0.50, 1.03)
Missing	6 (0.77)	1 (0.34)		

a OR adjusted for sex, age at enrolment, sex, country of birth, SEIFA and rural-urban residence
b OR adjusted for sex, age at enrolment, country of birth, SEIFA, rural-urban residence and birth weight

Table S4. Sensitivity analysis restricting CONFIRM controls to Victoria only for early life exposures and MM risk

Exposure	Cases, n (%)	Controls, n (%)	OR	(95% CI)
Maternal smoking in pregnancy[a]				
No	605 (77.4)	560 (75.9)	1.00	
Yes	91 (11.6)	73 (9.9)	1.13	(0.80, 1.61)
Missing	86 (11.0)	105 (14.2)		
Breastfeeding[a]				
No	98 (12.5)	109 (14.8)	1.00	
Yes	514 (65.7)	449 (60.8)	1.07	(0.79, 1.47)
Missing	170 (21.7)	180 (24.4)		
Height compared to other children at age 7[b]				
About the same	446 (57.0)	399 (54.1)	1.00	
Shorter	152 (19.4)	166 (22.5)	0.85	(0.65, 1.11)
Taller	175 (22.4)	148 (20.1)	1.06	(0.81, 1.39)
Missing	9 (1.2)	25 (3.4)		
Height compared to other children at age 11[b]				
About the same	409 (52.3)	361 (48.9)	1.00	
Shorter	161 (20.6)	171 (23.2)	0.86	(0.66, 1.13)
Taller	203 (26.0)	181 (24.5)	1.01	(0.78, 1.31)
Missing	9 (1.2)	25 (3.4)		
Time of growth spurt during teenage years[b]				
About the same	599 (76.6)	544 (73.7)	1.00	
Before most others	71 (9.1)	79 (10.7)	0.89	(0.62, 1.28)
After most others	94 (12.0)	83 (11.2)	1.03	(0.74, 1.44)
Missing	18 (2.3)	32 (4.3)		
Number of children you lived with (excluding yourself)[a]				
0	89 (11.4)	56 (7.6)	1.00	
1	155 (19.8)	146 (19.8)	0.76	(0.50, 1.16)
2	185 (23.7)	195 (26.4)	0.68	(0.45, 1.02)
3	160 (20.5)	134 (18.2)	0.92	(0.60, 1.41)
4	88 (11.3)	84 (11.4)	0.73	(0.46, 1.16)
5+	98 (12.5)	101 (13.7)	0.62	(0.40, 0.98)
Missing	7 (0.90)	22 (3.0)		
Shared a bedroom before 11 years old[a]				
No	208 (26.6)	155 (21.0)	1.00	
Yes	568 (72.6)	560 (75.9)	0.76	(0.59, 0.98)
Missing	6 (0.77)	23 (3.1)		
Contact with pets before 11 years old[a]				
No	175 (22.4)	107 (14.5)	1.00	
Yes	601 (76.9)	605 (82.0)	0.66	(0.50, 0.88)
Missing	6 (0.77)	26 (3.5)		

a OR adjusted for sex, age at enrolment, sex, country of birth and SEIFA
b OR adjusted for sex, age at enrolment, country of birth, SEIFA, and birth weight

Table S5. Sensitivity analysis for early life exposures and MM risk among EMMA cases and EMMA controls with additional adjustment for marital status

Exposure	Cases, n (%)	Controls, n (%)	OR	(95% CI)
Maternal smoking in pregnancy[a]				
No	605 (77.4)	224 (75.4)	1.00	
Yes	91 (11.6)	32 (10.8)	1.09	(0.69, 1.71)
Missing	86 (11.0)	41 (13.8)		
Breastfeeding[a]				
No	98 (12.5)	50 (16.8)	1.00	
Yes	514 (65.7)	197 (66.3)	1.19	(0.80, 1.77)
Missing	170 (21.7)	50 (16.8)		
Height compared to other children at age 7[b]				
About the same	446 (57.0)	152 (51.2)	1.00	
Shorter	152 (19.4)	68 (22.9)	0.78	(0.54, 1.12)
Taller	175 (22.4)	76 (25.6)	0.79	(0.55, 1.12)
Missing	9 (1.2)	1 (0.34)		
Height compared to other children at age 11[b]				
About the same	409 (52.3)	139 (46.8)	1.00	
Shorter	161 (20.6)	64 (21.5)	0.85	(0.59, 1.23)
Taller	203 (26.0)	93 (31.3)	0.74	(0.53, 1.04)
Missing	9 (1.2)	1 (0.34)		
Time of growth spurt during teenage years[b]				
About the same	599 (76.6)	227 (76.4)	1.00	
Before most others	71 (9.1)	33 (11.1)	0.94	(0.58, 1.50)
After most others	94 (12.0)	35 (11.8)	1.01	(0.64, 1.57)
Missing	18 (2.3)	2 (0.67)		
Number of children you lived with (excluding yourself [a])				
0	89 (11.4)	26 (8.8)	1.00	
1	155 (19.8)	71 (23.9)	0.72	(0.41, 1.25)
2	185 (23.7)	76 (25.6)	0.80	(0.46, 1.37)
3	160 (20.5)	49 (16.5)	1.13	(0.64, 2.01)
4	88 (11.3)	32 (10.8)	0.86	(0.46, 1.62)
5+	98 (12.5)	43 (14.5)	0.69	(0.38, 1.26)
Missing	7 (0.90)	0 (0.00)		
Shared a bedroom before 11 years old[a]				
No	208 (26.6)	76 (25.6)	1.00	
Yes	568 (72.6)	220 (74.1)	1.01	(0.73, 1.39)
Missing	6 (0.77)	1 (0.34)		
Contact with pets before 11 years old[a]				
No	175 (22.4)	54 (18.2)	1.00	
Yes	601 (76.9)	242 (81.5)	0.73	(0.50, 1.05)
Missing	6 (0.77)	1 (0.34)		

a Sex, age at enrolment, sex, country of birth, SEIFA, rural-urban residence and marital status
b Sex, age at enrolment, country of birth, SEIFA, rural-urban residence, birth weight and marital status

Table S6. Sensitivity analysis restricting CONFIRM controls to Victoria only for early life exposures and MM risk with additional adjustment for marital status

Exposure	Cases, n (%)	Controls, n (%)	OR	(95% CI)
Maternal smoking in pregnancy[a]				
No	605 (77.4)	560 (75.9)	1.00	
Yes	91 (11.6)	73 (9.9)	1.15	(0.81, 1.64)
Missing	86 (11.0)	105 (14.2)		
Breastfeeding[a]				
No	98 (12.5)	109 (14.8)	1.00	
Yes	514 (65.7)	449 (60.8)	1.06	(0.77, 1.45)
Missing	170 (21.7)	180 (24.4)		
Height compared to other children at age 7[b]				
About the same	446 (57.0)	399 (54.1)	1.00	
Shorter	152 (19.4)	166 (22.5)	0.86	(0.66, 1.13)
Taller	175 (22.4)	148 (20.1)	1.09	(0.83, 1.43)
Missing	9 (1.2)	25 (3.4)		
Height compared to other children at age 11[b]				
About the same	409 (52.3)	361 (48.9)	1.00	
Shorter	161 (20.6)	171 (23.2)	0.86	(0.66, 1.13)
Taller	203 (26.0)	181 (24.5)	1.04	(0.80, 1.35)
Missing	9 (1.2)	25 (3.4)		
Time of growth spurt during teenage years[b]				
About the same	599 (76.6)	544 (73.7)	1.00	
Before most others	71 (9.1)	79 (10.7)	0.90	(0.62, 1.30)
After most others	94 (12.0)	83 (11.2)	1.03	(0.74, 1.45)
Missing	18 (2.3)	32 (4.3)		
Number of children you lived with (excluding yourself)[a]				
0	89 (11.4)	56 (7.6)	1.00	
1	155 (19.8)	146 (19.8)	0.81	(0.53, 1.23)
2	185 (23.7)	195 (26.4)	0.73	(0.48, 1.10)
3	160 (20.5)	134 (18.2)	0.97	(0.63, 1.49)
4	88 (11.3)	84 (11.4)	0.76	(0.48, 1.22)
5+	98 (12.5)	101 (13.7)	0.65	(0.41, 1.02)
Missing	7 (0.90)	22 (3.0)		
Shared a bedroom before 11 years old[a]				
No	208 (26.6)	155 (21.0)	1.00	
Yes	568 (72.6)	560 (75.9)	0.78	(0.61, 1.00)
Missing	6 (0.77)	23 (3.1)		
Contact with pets before 11 years old[a]				
No	175 (22.4)	107 (14.5)	1.00	
Yes	601 (76.9)	605 (82.0)	0.68	(0.51, 0.90)
Missing	6 (0.77)	26 (3.5)		

a OR adjusted for age, sex, age at enrolment, sex, country of birth, SEIFA and marital status
b OR adjusted for sex, age at enrolment, country of birth, SEIFA, birth weight and marital status
